# Virulence, Susceptibility Profile, and Clinical Characteristics of Pathogenic Coagulase-Negative Staphylococci

**DOI:** 10.7759/cureus.66397

**Published:** 2024-08-07

**Authors:** Rhea Michelle J Khodabux, Shanthi Mariappan, Uma Sekar

**Affiliations:** 1 Microbiology, Sri Ramachandra Institute of Higher Education and Research, Chennai, IND

**Keywords:** methicillin-resistant coagulase-negative staphylococcus, coagulase-negative staphylococci, antimicrobial resistance (amr), virulence genes, sccmec typing

## Abstract

Background

Coagulase-negative staphylococci (CoNS) are emerging as clinically significant pathogens. A high proportion of methicillin resistance along with intense biofilm-producing ability render CoNS-related infections challenging to treat. This study was undertaken to investigate the mechanisms of methicillin resistance, identify genes encoding for virulence, and their association with clinical outcomes among clinical isolates of Staphylococci in a tertiary care center.

Methods

A total of 203 clinical isolates were included in this study. Susceptibility to various antibiotics was determined by the disc diffusion method. Methicillin resistance was screened using cefoxitin disc, *mecA* and *mecC* genes were detected using polymerase chain reaction (PCR). PCR was performed to detect five virulence genes: *atlE*, *aap*, *fbe*, *embp,* and *icaAB*. Staphylococcal cassette chromosome mec (SCCmec) types were identified by multiplex PCR. Statistical analysis was performed using SPSS software (IBM Inc., Armonk, New York). The Chi-squared test was used to compare the distribution of virulence genes among methicillin-susceptible resistant CoNS. A p-value of less than 0.5 was considered significant.

Results

In the current study, 60% (122/203) of CoNS were methicillin-resistant, and SCCmec type I was the most common. Among the 203 CoNS, 24.6% (50/203) isolates harbored one or more virulence genes in them.

Conclusion

CoNS have relatively low virulence as only 24.6% of isolates carried the virulence genes. Nevertheless, the variety of diseases linked to these species indicates the necessity for accurate identification and precise reporting of antimicrobial susceptibility to avoid adverse outcomes.

## Introduction

Coagulase-negative staphylococci (CoNS) form the microbiota of the skin and mucous membrane of humans. CoNS have emerged as clinically significant pathogens in about 12 to 25% of cases, especially in critically ill, long-term hospitalized, immunocompromised patients and in those harboring invasive medical devices [1]. *Staphylococcus epidermidis* is the most common pathogenic species. Other pathogenic CoNS species include *Staphylococcus saprophyticus*, *Staphylococcus haemolyticus,* and *Staphylococcus hominis* [2]. The pathogenicity of CoNS species is attributed to the possession of a host of virulence factors that aid in the establishment of infection within the host. Therapeutically, CoNS infections are challenging due to a large proportion of methicillin-resistant strains and increasing numbers of isolates with reduced susceptibility to glycopeptides and oxazolidinones [3]. The accurate identification of CoNS and distinct differentiation between clinically significant and contaminant bacteria is important in establishing their role in infections [4].

The ability to form biofilms is considered the most significant virulence factor of CoNS, particularly in the context of infections associated with medical devices like catheters and prosthetic implants. Biofilms are complex communities of bacterial cells enclosed in a self-produced matrix. This matrix provides CoNS with several advantages, including protection against the host immune system and resistance to antibiotics. The process of biofilm formation in CoNS unfolds through several stages, such as initial attachment, accumulation, maturation, and detachment. The first stage of biofilm formation takes place via the proteins present in the bacterial cell wall autolysin E (*atlE*), and fibrinogen binding protein (*fbe/sdrG*). It has recently been proposed that *sdrG *can bind to host cells such as osteoblasts and colonize implants [5, 6].

The accumulative stage is characterized by the production of polysaccharide intercellular adhesin (PIA), also known as poly N-acetyl glucosamine (PNAG), a common adhesive molecule. PNAG is synthesized by enzymes encoded by the intercellular adhesion (*ica*) locus, which includes the genes *icaA*, *icaD*, *icaB*, and *icaC*, as well as the regulatory gene *icaR*, which is positioned upstream of the *icaADBC* and thus divergently transcribed [7]. PIA-independent biofilm formation in CoNS is by the cell surface-associated proteins, namely accumulation-associated protein (*aap*) and extracellular matrix-binding protein (*embp*), that mediate bacterial binding to fibronectin, and also participate in biofilm accumulation.

Treatment of methicillin-resistant CoNS (MRCoNS) infections contributes to high treatment costs and increased morbidity and mortality [3]. The possession of multidrug-resistance (MDR) determinants on mobile plasmids facilitates the transfer of these elements among CoNS species and to *Staphylococcus aureus *[8].

In India, there is a paucity of information regarding the virulence profile of pathogenic coagulase-negative *Staphylococci*, as well as their association with the clinical spectrum of infections and antimicrobial resistance. The outcomes of infections due to the *Staphylococcus *species are also poorly understood in relation to the genotype. Hence, this study was undertaken to investigate the mechanisms of methicillin resistance, detect the genes encoding for virulence, and examine their association with clinical outcomes among clinical isolates of CoNS in a tertiary care center.

## Materials and methods

Bacterial isolates

The study was conducted in a 1600-bed university teaching hospital in South India from 2019 to 2021. To determine the appropriate sample size for our study, we utilized the prevalence estimate obtained from a prior investigation conducted in South India, wherein the prevalence of the virulence genes among CoNS was 15.1% [6]. For a prevalence of 15%, with a 95% confidence level and a margin of error of ±5%, the sample size required was calculated as 196. In consideration of the potential loss of viability during storage, we collected a few additional isolates. In the current study, a total of 203 clinically significant, consecutive, non-repetitive coagulase-negative *Staphylococci *were included. Repetitive isolates from the same patients and isolates from outpatients were excluded from the study.

The source of the isolates was exudates (n=95), namely pus, fluids, aspirates, blood (n=95), and urine (n=13). The clinical significance of the study isolates was ascertained by correlating with gram stain, simultaneous isolation from multiple samples, significant growth in cultures, and the patient's clinical history. The clinical history of all the patients was collected from the medical records department. The isolates were identified up to species level by standard biochemical tests such as coagulase test, production of urease, fermentation of 1% urease, and automated systems: VITEK2 GP-card (bioMerieux, Marcy l'Etoile, France), and MALDI-TOF MS (bioMerieux, Marcy l'Etoile, France).

Antimicrobial susceptibility testing

Antibiotic susceptibility testing was performed using the Kirby-Bauer disc diffusion technique. The discs used in the current study were ampicillin (10µg), erythromycin (30µg), clindamycin (2µg), gentamicin (30µg), ciprofloxacin (5µg) and linezolid (30µg). Methicillin resistance was detected by using a cefoxitin (30µg) disc (Himedia, Mumbai, Maharashtra, India) as per Clinical and Laboratory Standards Institute (CLSI) guidelines (CLSI-M100-S29) [9]. The minimum inhibitory concentration (MICs) of linezolid (MicroExpress, Goa, India), teicoplanin, and vancomycin were determined using the agar dilution method in accordance with CLSI guidelines.

Phenotypic methods

Production of Biofilm: Microtiter Plate Method (MTP)

Biofilm production was determined by the microtiter plate method (MTP) utilizing a 96-well polystyrene microtiter plate [10]. Colonies of *Staphylococci *were inoculated in trypticase soy broth enriched with glucose and incubated overnight. Two hundred μl of the overnight broth was added into each well of the microtiter plate and re-incubated overnight. The contents of the well were then discarded. Each well was washed with sterile 200 μl phosphate buffer saline and then discarded. The optical density was noted. The isolates were classified as strong biofilm producers with an OD of >0.240, moderate biofilm producers with an OD of 0.120-0.240, and non-biofilm producers with an OD of <0.120.

Molecular methods

DNA Extraction

DNA extraction was performed using the boiling-lysis method as described previously [11, 12]. A loopful of overnight *Staphylococcal *culture was added to 250 μl of sterile MiliQ water. It was placed in a dry bath at 100°C for 10 minutes and then immediately placed in -20°C for five minutes. It was then thawed and centrifuged at 12,000 rpm for 10 minutes. Two μl of this was then used as a template in all the PCR reactions.

Polymerase Chain Reaction (PCR)

*Staphylococcus epidermidis* and *Staphylococcus haemolyticus* were subjected to molecular confirmation using the species-specific *nuc* gene [13]. *MecA* and *mecC* genes were amplified to detect methicillin resistance [14, 15].

Duplex and simplex PCR were set up to detect the virulence genes with the following PCR conditions: initial denaturation at 94°C for 5min, denaturation at 94°C for 15 s, annealing: variable for 30 s, extension at 72°C for 35 cycles for 30 s, final extension at 72°C for 45 s. All the PCR reactions were carried out with a final volume of 25 µl reaction. Multiplex PCR was set up for Staphylococcal cassette chromosome mec (SCCmec) typing. The PCR conditions for SCCmec typing were adjusted according to a previously described method [12, 16]. The amplicons were separated in a 2% agarose gel containing ethidium bromide. The primers used are described in Table 1. Sequenced strains were used as positive controls and sterile Mili Q water was used as negative control [6]. 

**Table 1:** List of primers employed in the study

Gene	Primer	Amplicon
Primers for species identification		
nuc- Staphylococcus haemolyticus	F- TAGTGGTAGGCGTATTAGCC	434 bp
	R- ACGATATTTGCCATTCGGTG	
nuc- Staphylococcus epidermidis	F- TTGTAAACCATTCTGGACCG	251 bp
	R- ATGCGTGAGATACTTCTTCG	
Primers for methicillin resistance		
mecA	F- GTAGAAATGACTGAACGTCCGATAA	310 bp
	R- CCAATTCCACATTGTTTCGGTCTAA	
mecC	F- GAAAAAAAGGCTTAGAACGCCTC	138 bp
	R- GAAGATCTTTTCCGTTTTCAGC	
Primers for virulence factors of coagulase-negative *Staphylococci*		
aap	F- AAACGGTGGTATCTTACGTGAA	466 bp
	R- CAATGTTGCACCATCTAAATCAGCT	
fbe	F- CTACAAGTTCAGGTCAAGGACAAGG	273 bp
	R- GCGTCGGCGTATATCCTTCMG	
atlE	F- CAACTGCTCAACCGAGAACA	682 bp
	R- TTTGTAGATGTTGTGCCCCA	
embp	F- AGCGGTACAAATGTCAAT	455 bp
	R- AGAAGTGCTCTAGCATCATCC	
icaAB	F- TTATCAATGCCGCAGTTGTC	516 bp
	R- GTTTAACGCGAGTGCGCTAT	
Primers for *Staphylococcal *cassette chromosome mec (SCCmec) typing		
β	F- ATTGCCTTGATAATAGCCYTCT	937 bp
a3	R- TAAAGGCATCAATGCACAAACACT	
ccrC	F- CGTCTATTACAAGATGTTAAGGATAAT	518 bp
ccrC	R- CCTTTATAGACTGGATTATTCAAAATAT	
1272	F- GCCACTCATAACATATGGAA	415 bp
1272	R- CATCCGAGTGAAACCCAAA	
5RmecA	F- TATACCAAACCCGACAACTAC	359 bp
5R431	R- CGGCTACAGTGATAACATCC	

DNA Sequencing

Positive PCR products were sequenced. Sangers DNA sequencing was performed using the ABI 3730XL sequencer (Applied Biosystems, Waltham, Massachusetts) with the ABI PRISM BigDye Terminator.

Statistical analysis

Statistical analysis was performed using SPSS software 16.0 version (IBM Inc., Armonk, New York). The Chi-squared was performed to compare the distribution of virulence genes among the methicillin-resistant CoNS (MRCoNS) and methicillin-susceptible CoNS (MSCoNS). Univariate analysis was performed to compare the association between mortality and virulence factors of Staphylococci. A p-value of less than 0.05 was considered as statistically significant.

## Results

Bacterial isolates

Among the CoNS, 14 different species were identified. *Staphylococcus haemolyticus* (n=84) was the most common, followed by *Staphylococcus hominis* (n=50) and *Staphylococcus epidermidis* (n=39). *Staphylococcus cohnii *(n=7), *Staphylococcus saprophyticus* (n=4), *Staphylococcus sciuri *(n=4), *Staphylococcus capitis* (n=4), *Staphylococcus xylosus* (n=3), *Staphylococcus arlettae* (n=3), *Staphylococcus hyicus* (n=1), *Staphylococcus intermedius* (n=1), *Staphylococcus pasteurii* (n=1), *Staphylococcus simulans* (n=1), and *Staphylococcus warneri *(n=1) were isolated less frequently.

Antimicrobial susceptibility pattern

The antibiotic susceptibility pattern is as follows: ampicillin (13.3%), erythromycin (27%), clindamycin (55.1%), ciprofloxacin (46.3%), gentamicin (70%), cefoxitin (40%), and linezolid (98.5%). Linezolid resistance was detected in three isolates of *Staphylococcus haemolyticus* with a MIC of >128µg/ml. All the isolates were susceptible to vancomycin and teicoplanin.

Biofilm production by microtiter plate method

Among the 203 CoNS, 120 isolates produced biofilm, while 83 isolates did not produce biofilm. Among the 120 biofilm producers, 117 were moderate biofilm producers, whereas three produced strong biofilm.

Genotypic methods

Species Identification

The *nuc* gene was detected in all the *Staphylococcus haemolyticus* (n=84) and *Staphylococcus epidermidis* (n=39), confirming their species.

Detection of Methicillin Resistance

The *MecA* gene was detected in all 122 MRCoNS isolates. Of the 14 species of CoNS, only 12 species exhibited resistance to cefoxitin and carried the *mecA* gene. *Staphylococcus hyicus *(n=1) and *Staphylococcus intermedius* (n=1) did not harbor the *mecA* and were susceptible to methicillin. The *mecC* gene was not detected in any of the study isolates.

SCCmec Typing

Among the 122 MRCoNS, the most predominant type was SCCmec type I at 58% (71/122). Others belonged to types such as III in 8.1% (10/122), IV in 6.5% (8/122), V in 1.6% (2/122), and II in 0.8% (1/122). The coexistence of two SCCmec elements, I and III, were seen in thee isolates, and SCCmec type III and IV were found in a single isolate. Twenty-six isolates were non-typeable.

Detection of virulence genes of coagulase-negative *Staphylococci *by PCR

**Figure 1:**
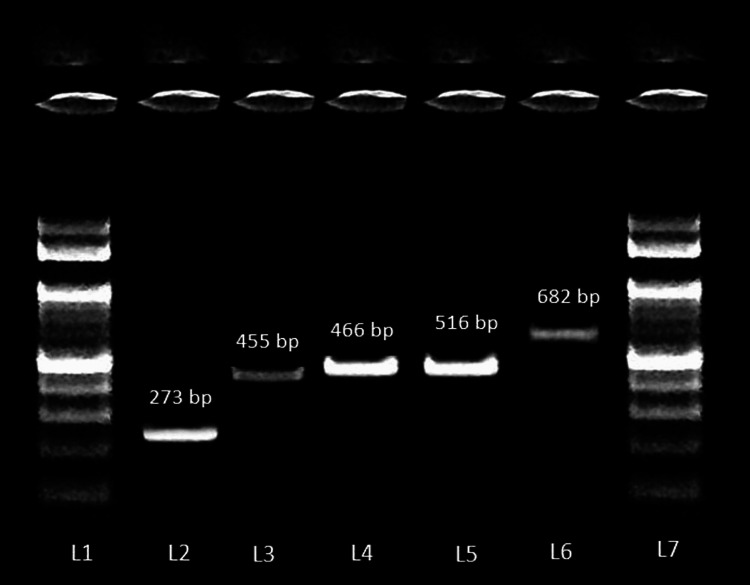
Gel electrophoresis image of the virulence genes of coagulase-negative Staphylococci detected by polymerase chain reaction Lane 1 and 7 - 100 bp ladder; Lane 2 - *fbe *(273 bp); Lane 3 - *embp *(455 bp); Lane 4 - *aap *(466 bp); Lane 5 - *icaAB *(516 bp); Lane 6 - *atlE *(682 bp)

Among the 203 CoNS, 24.6% (50/203) isolates carried one or more virulence genes. The most common genes detected in this study were *atlE *(33/203), *embp *(30/203), *fbe *(27/203; Table 2). One hundred and fifty-three (75.3%) study isolates did not harbor any of the genes that were looked for in this study. Some genes occurred singly in 28% (14/50) of the study isolates. The virulence gene determinants that occurred singly were *icaAB *(n=5), *atlE *(n=4), *fbe *(n=3), *aap *(n=1), and *embp *(n=1). Figure 1 depicts the virulence genes of coagulase-negative *Staphylococci*.

*Staphylococcus epidermidis* harbored more virulence genes than *Staphylococcus haemolyticus* which was the most common species detected in the study. Some of the CoNS did not harbour any of the five common genes looked for in the study which included, *Staphylococcus saprophyticus*, *Staphylococcus sciuri*, *Staphylococcus xylosus*, *Staphylococcus arlettae*, *Staphylococcus hyicus*, *Staphylococcus intermedius*, *Staphylococcus simulans*, and *Staphylococcus warneri*.

The MSCoNS carried more virulence genes when compared to MRCoNS. Of the five virulence genes looked for, the *atlE*, *embp*, and *fbe *were more prevalent in MSCoNS than in MRCoNS. Their distribution was statistically significant (Table 2).

**Table 2:** Distribution of the virulence genes of coagulase-negative Staphylococci *Chi-squared test was used for statistical analysis

Virulence genes	Total	Percentage	MRCoNS (n=122)	Percentage	MSCoNS (n=81)	Percentage	P-value^*^
atlE	33	16.2%	9	7.3%	24	29.6%	0.000026
embp	30	14.77%	10	8.1%	20	24.6%	0.001183
fbe	27	13.3%	11	9.1%	16	19.7%	0.027385
icaAB	17	8.37%	9	7.3%	8	9.8%	0.528978
aap	14	6.8%	6	4.9%	8	9.8%	0.17216

Among the blood isolates, *atlE* was the most predominant gene detected, followed by *embp*, *fbe* and *icaAB*. Among the exudative specimens, *atlE* followed by *embp* and *fbe*, were more common. Thirty isolates anchored more than one gene. The combination of *fbe*,* atlE*, and* embp* was the most common. Multiple virulence gene combinations in single isolates were more common in MSCoNS (n=19) than in MRCoNS (n=11). (Table *3*)

**Table 3:** Virulence gene combinations in coagulase-negative Staphylococci MSCoNS - methicillin-susceptible coagulase-negative staphylococci

Virulence genes in single isolates				
Virulence genes			MSCoNS (n = 6)	
aap, fbe, embp			1	
icaAB, aap			1	
icaAB, fbe, atlE			1	
icaAB, atlE,			1	
icaAB, atlE, fbe			1	
icaAB, embp			1	
Virulence genes in multiple isolates				
Virulence genes	Number of isolates (n = 30)	MRCoNS (n = 11)		MSCoNS (n = 19)
fbe, atlE, embp	10	3		7
atlE, embp	9	2		7
aap, fbe, atlE, embp	3	-		3
icaAB, aap, fbe, atlE, embp	2	2		-
icaAB, aap, fbe, atlE	2	1		1
aap, fbe, embp	2	2		-
fbe, aap	2	1		1
Single virulence genes in multiple isolates				
Virulence genes	Number of isolates (n = 14)	MRCoNS (n = 8)		MSCoNS (n = 6)
icaAB	5	4		1
atlE	4	1		3
fbe	3	2		1
aap	1	-		1
embp	1	1		-

A mortality rate of 21.6% (44/203) was observed among the patients with CoNS infection, which included (28/44) MRCoNS and (16/44) MSCoNS. Fifteen isolates from these patients carried two or more virulence genes. Notably, 29 isolates did not carry any of the virulence determinants. Univariate analysis of the mortality-associated virulence gene revealed a significant association between mortality and the presence of virulence gene *icaAB* (Table 4).

**Table 4:** Univariate analysis between mortality-associated virulence factors of coagulase-negative Staphylococci *Chi-squared test was used for statistical analysis

Virulence genes	Present/ absent	Deceased (n=44)	Survivors (n=159)	Odds ratio 95% confidence interval	p-value*
icaAB	Present	7	9	3.1532 (1.1021- 9.0217)	0.0323
	Absent	37	150		
aap	Present	6	8	2.9803 (0.9756-9.1030)	0.0553
	Absent	38	151		
fbe	Present	8	19	1.6374 (0.6633-4.0421)	0.2848
	Absent	36	140		
altE	Present	10	23	1.7391 (0.7567-3.9968)	0.1924
	Absent	34	136		
embp	Present	7	23	1.1187 (0.4455-2.8094)	0.1924
	Absent	37	136		

DNA sequencing 

The obtained sequences were submitted in the GenBank database under the following accession numbers: OQ451858 (*aap*), OQ451859 (*fbe*), OQ451860 (*icaAB*), OQ555810 (*atlE*), OQ572692 (*embp*), ON249039 (*mecA*), ON249040 (*Staphylococcus haemolyticus nuc*), OQ572693 (*Staphylococcus epidermidis nuc*)

## Discussion

In the present study, 203 CoNS isolates belonged to 14 different species. Among these, *Staphylococcus haemolyticus* (84/203) was the predominant species, followed by *Staphylococcus hominis* (50/203) and *Staphylococcus epidermidis* (39/203). The remaining 30 isolates belonged to 11 different species. The species distribution of CoNS in our study is in concordance with previous studies from India, where *Staphylococcus haemolyticus* was the most common, followed by *Staphylococcus epidermidis* [16, 17]. However, it contrasted with findings from other regions. For instance, in studies, *Staphylococcus epidermidis *was reported as the most prevalent CoNS species [18, 19]. A study from China reported *Staphylococcus hominis* as the most predominant species, but in the present study, it was the second most common, accounting for 24.6% [20]. Although once considered as skin colonizer with minimal virulence potential, *Staphylococcus hominis *is now a notable coagulase-negative *Staphylococcus *(CoNS) with clinical pathogenic potential in hospital settings.

The majority of *Staphylococcus haemolyticus* isolates were obtained from exudative specimens, followed by blood and urinary specimens. *Staphylococcus hominis *were predominantly isolated form wound specimens, whereas *Staphylococcus epidermidis* was frequently from blood samples. *Staphylococcus saprophyticus* is known to be associated with urinary tract infections, but in the present study, it was obtained from exudative and blood specimens. 

The diversity of CoNS species identified in our study, with 14 different species, reflects their complex and varied nature. This variation can be attributed to the difference in colonization characteristics of study patients, geographical factors, and the varying adaptability of different species to selective pressures such as biocides and antimicrobials in the environment.

In the present study, methicillin resistance was observed in 60% (122/203) of the CoNS. The *mecA* determinant was carried by all the methicillin-resistant CoNS (MRCoNS). The prevalence of MRCoNS was in alignment with earlier studies from India, ranging from 48.2% to 60% [16, 17, 19]. However, it is lower when compared to studies from Iran and Turkey, where the frequency of MRCoNS was found to be 68.6% and 83.3%, respectively [21]. Methicillin resistance also varied among different species of CoNS. Among the 14 species of CoNS in this study, 12 species exhibited methicillin resistance. The highest rates of methicillin resistance in the current investigation were found in *Staphylococcus cohnii *(85.7%), followed by *Staphylococcus haemolyticus *(78.5%), *Staphylococcus xylosus* (66.7%), *Staphylococcus sciuri* (50%), *Staphylococcus epidermidis* (46%), and *Staphylococcus hominis* (44%).

In the current study, among the MRCoNS (122/203), the predominant SCCmec type was type I (71/122). The other types encountered were types III (10/122), IV (8/122), V (2/122), and II (1/122). This is concordant with other studies [16, 22]. The carriage of more than one type of SCCmec element is not an uncommon feature in MRCoNS. This finding is evident in the present study, where the coexistence of two SCCmec elements, I and III, were seen in three isolates and SCCmec type II and IV in a single isolate. This analogous pattern of coexistence of SCCmec types has been previously cited [11, 16, 22]. Furthermore, 26/122 isolates were non-typable, which can be attributed to the presence of SCCmec genes other than types I to V. A study from Thailand reported that 56.1% (23/41) were untypable [2]. Varied distribution of SCCmec types in MRCoNS can be influenced by host species and regional variances. The presence of numerous *ccr* gene complexes, differences in *ccr* gene amplification, and new combinations of *mec* and *ccr* complexes make SCCmec typing difficult and may give inaccurate results. Application of pulse field gel electrophoresis and multilocus sequence typing can yield clear results [1]. This study did not employ these typing methods.

An overall high prevalence of resistance to all antibiotics was seen among CoNS, with higher resistance to non-β-lactam antimicrobials such as erythromycin (73%), ciprofloxacin (53.7%), clindamycin (44.9%) and gentamicin (30%). These findings are comparable to the resistance profile reported by other authors from India [16, 17, 19]. Clearly, these antibiotics no longer remain as options for empirical treatment of CoNS infections. All the study isolates were susceptible to vancomycin. Three isolates of *Staphylococcus hemolyticus* exhibited resistance to linezolid with MIC of 128μg/ml.

In the current study, biofilm production was detected by the microtiter plate method in 59.2% (120/203). This finding is comparable to previous studies that have noted the frequency of biofilm development in clinical CoNS isolates to be ranging from 40.9% to 75.3% [23, 24]. Of the 120 biofilm producers, three produced strong biofilm, and 117 produced moderate biofilms.

The presence of five common virulence genes *altE*, *fbe*, *icaAB*, *aap*, *embp* was investigated. The presence of one or more virulence genes was observed in 24.6% (50/203). All the five genes were detected predominantly among *Staphylococcus epidermidis*. The bifunctional autolysin encoding *altE* (16.2%, 33/203) was the most common gene, followed by the extracellular matrix binding protein coding *embp* (14.7%, 30/203). The fibrinogen binding protein encoding *fbe* was harbored by 13.3% (27/203), *icaAB* in 8.37 %(17/203), and *aap* in 6.8% (13/203). Thirty isolates anchored more than one gene in single isolates. The combination of *fbe*, *atlE,* and *embp* was the most common. Occurrences of more than one gene were more common in MSCoNS (n=19) than in MRCoNS (n=11). An Indian study described the prevalence of virulence genes among biofilm producers. The study reported that, of the 17 strong and 24 moderate biofilm-forming CoNS screened genotypically, only nine strong and fourteen moderate CoNS were found to be positive for either single or multiple biofilm genes [6]. Studies from Brazil and Kuwait have reported an overall prevalence of 42% and 61.3% respectively [25, 26].

On comparing biofilm production and occurrence of the virulence gene, we observed that one or more of these genes were present in the moderate biofilm producers. Of the 50 isolates that carried the virulence genes, 28 were moderate biofilm producers. A proportion of non-biofilm producers (22/50) also anchored these genes. It has been previously reported that even in the presence of *ica *genes or the other biofilm-encoding genes, strains may not form a biofilm in vitro due to the non-expression of these genes [25]. Among three strong biofilm producers, none of these genes were present, indicating that biofilm production is probably encoded by other genes such as *icaC*, *icaD*, *bap*, and *bhp*, which were not included in the study protocol.

There was a significant difference in the distribution of virulence genes such as fibrinogen binding protein *fbe*, autolysin *atlE,* and extracellular matrix binding protein *embp* among MRCoNS and MSCoNS. In the current study, MSCoNS harbored more virulence genes compared to MRCoNS, suggesting that MSCoNS remains a substantial cause of infection and that MRCoNS has not replaced MSCoNS. Studies comparing virulence gene distribution among MRCoNS and MSCoNS are limited. Few available studies indicate that methicillin resistance was found among biofilm producers in comparison with non-biofilm producers. [27]

A mortality rate of 21.6% (44/203) was observed among the patients with CoNS infection, which included (28/44) MRCoNS and (16/44) MSCoNS. Although 44 patients had fatal outcomes, infections caused by CoNS cannot be solely attributed to the outcome. Univariate analysis of mortality-associated virulence gene exhibited a significant association betwen mortality and the presence *icaAB*. The association of virulence factors with morbidity cannot be explicitly ascertained due to the presence of several comorbid conditions such as diabetes mellitus, hypertension, coronary artery disease, chronic kidney disease, and multiorgan dysfunction. These patients also had indwelling devices such as vascular catheters, prosthetic devices, urinary catheter, and a few had undergone surgical procedures. Among the 44 patients who had fatal outcomes, 34 CoNS were isolated from blood specimens of patients with sepsis, six from exudative specimens of patients with diabetic ulcers, and four were urinary isolates. Of the 44 isolates obtained from these patients, strong biofilm was produced by one isolate, and 19 produced moderate biofilms. Furthermore, 15 isolates carried one or more virulence genes, with *atlE* and *embp* being the most common. Of the 159 isolates from patients who had recovered, biofilm was produced by 100 isolates. Thirty-five isolates carried virulence genes in different combinations. Thus, biofilm production and virulence genes occurred in both subsets of patients who recovered or had fatal outcomes. Hence, an association between the presence of virulence genes, type of infection, and clinical outcome could not be established.

Strength

The strength of this study is the characterization of five common virulence determinants among 203 clinically significant pathogenic CoNS. Comprehensive species identification of 14 different species of CoNS, analysis of their association with the type of infection, antimicrobial susceptibility pattern, and clinical characteristics of the source patients are the additional assets of this study.

Limitations

A comparison of the virulence and antimicrobial resistance profile between pathogenic and commensal CoNS was not done since the study included only the pathogenic CoNS isolates. Virulence genes specific for each species of CoNS were not analyzed in the present study due to the scarce number of isolates in several of the CoNS species.

## Conclusions

CoNS retain their pathogenic potential despite the fact that only 24.6% of isolates carried the virulence genes. These organisms are increasingly recognized as clinically significant, and a multitude of infections are being attributable to them. This is in alignment with the virulence trade-off hypothesis, which predicts that an intermediate level of virulence supports evolution. As the pathogenic significance becomes apparent, it becomes necessary to characterize them and study their antimicrobial susceptibility profile. Because CoNS are usually considered part of normal skin flora, most clinical laboratories do not test for antimicrobial susceptibility unless from a sterile site such as blood. There is a marked species diversity among CoNS. The virulence genes specific to various species of CoNS must be investigated further, which will give better insights into the pathogenic mechanisms involved. Variation in their virulence and antimicrobial susceptibility profile calls for accurate speciation.

## Appendices

**Table 5:** Mortality associated virulence genes and clinical profile of the patients with CoNS-related infections DM- diabetes mellitus, CKD- chronic kidney disease, CRBSI- catheter related blood stream infection, CAD- coronary artery disease, VAP- ventilator acquired pneumonia, MODS- multiorgan dysfunction syndrome, CVA- cerebrovascular accident, UGI bleed- upper gastrointestinal tract, DCLD- decompensated chronic liver disease, RTA-road traffic accident, UTI- urinary tract infection, HTN- hypertension, A- ampicillin, CD- clindamycin, E- erythromycin, CIP- ciprofloxacin, CXM- cefuroxime, G- gentamicin, LZ- linezolid, VA- vancomycin

Specimen	Virulence genes	Diagnosis	MRCONS / MSCONS	Antibiotic susceptibility
Staphylococcus haemolyticus				
Blood	*atlE*, *embp*	CKD, sepsis, perianal abscess	MSCONS	CD,G,CX,VA,LZ
Blood	*icaAB*, *atlE*	Multiple myeloma, CKD, sepsis	MSCONS	A,E,VA,LZ,CX
Blood	None	Sepsis, HTN, VAP	MSCONS	CX,VA,LZ
Exudate	*icaAB*, *aap*	Traumatic ulcer, wound infection following RTA, DM, HTN	MSCONS	CIP,CX,G,VA,LZ
Urine	None	DM, CVA, seizure	MRCONS (SCCmec type I)	G,VA,LZ
Exudate	None	Cellulitis, right hip implant infection	MRCONS (SCCmec type I)	VA,LZ
Blood	None	Sepsis, CKD, HTN, DM, CVA	MRCONS (SCCmec type I)	CD,VA,LZ
Urine	None	CKD, MODS, DM	MRCONS (SCCmec type I)	G,VA,LZ
Blood	None	Cellulitis, Sepsis, CKD, MODS	MRCONS (SCCmec type I)	CD,VA,LZ
Blood	None	Sepsis, UGI bleed, DCLD, CKD	MRCONS (SCCmec type I)	VA,LZ
Blood	None	Sepsis, CAD, CKD	MRCONS (SCCmec type I)	G,VA,LZ
Exudate	None	DM, HTN, CAD, CKD	MRCONS (SCCmec type I)	VA,LZ
Urine	None	CVA, sepsis, DM	MRCONS (SCCmec type I)	CIP,G,VA,LZ
Blood	*aap*, *fbe*	Duodenal ulcer, sepsis, DM, HTN, CKD	MR CONS (SCCmec type I)	CIP,G,VA,LZ
Blood	None	DM, CKD, sepsis	MR CONS (SCCmec type I)	VA, LZ
Blood	None	Urosepsis, DM, HTN, CKD	MR CONS (SCCmec type I)	G, VA, LZ
Blood	None	CKD, HTN, Sepsis	MR CONS (SCCmec type I)	E, CD, CIP, VA, LZ
Staphylococcus epidermidis				
Blood	*fbe*, *atlE*, *embp*	DM, urosepsis, CKD	MSCONS	E,CD,CIP,G,CX,VA,LZ
Blood	*atlE*, *embp*	Sepsis, UTI, cellulitis, CKD	MSCONS	E,CD,CIP,G,CX,VA,LZ
Blood	*icaAB*, *aap*, *fbe*	CAD, DM, VAP, CKD, sepsis	MSCONS	A,E, CD, CIP, G, VA, LZ, CTX
Blood	None	CVA, HTN, Sepsis	MRCONS (SCCmec non typable)	CD, VA, LZ
Blood	None	MODS, sepsis, Pyelonephritis, CVA, DM	MRCONS (SCCmec type I)	CD,G,VA,LZ
Blood	*icaAB*, *atlE*, *fbe*, *aap*, *embp*	DM, CAD, VAP, sepsis	MRCONS (SCCmec type III)	VA,LZ
Blood	*fbe*, *atlE*, *embp*	DM, connective tissue disorder, sepsis	MR CONS (SCCmec type I)	E, CD, CIP, G, VA, LZ
Urine	None	DM, HTN, CKD, sepsis	MR CONS (SCCmec type I)	G, VA, LZ
Blood	None	DM	MR CONS (SCCmec type III)	CD, CIP, G, VA, LZ
Staphylococcus hominis				
Blood	None	Sepsis, DM	MSCONS	CX,VA,LZ
Exudate	None	Sepsis, CKD,	MSCONS	CD,CIP,CX,VA,LZ
Exudate	fbe	Chronic Osteomyelitis	MSCONS	CD,G,CX,VA,LZ
Blood	*icaAB*, *aap*, *fbe*	DM, HTN, sepsis	MSCONS	E,CD,CIP,G,VA,LZ CTX
Blood	*atlE*, *embp*	Abscess, DM, sepsis	MSCONS	E, CD, G, VA, LZ, CTX
Exudate	None	DM, HTN	MSCONS	A, E, CD, G, VA, LZ, CTX
Blood	None	Septic shock multiorgan failure, VAP	MRCONS (SCCmec type III)	CD,VA,LZ
Blood	*icaAB*, *fbe*, *atlE*	Sepsis, supraglottic carcinoma	MRCONS (SCCmec type I)	E,CD,CIP,G,VA,LZ
Blood	*atlE*, *embp*	DM, cellulitis, sepsis	MRCONS (SCCmec non- typable)	G, VA, LZ
Blood	None	DM, UTI, sepsis	MRCONS (SCCmec type I)	CD, CIP, E, CIP, G, VA, LZ
Staphylococcus cohnii				
Blood	None	CAD, MODS, DM, AKI, UTI, sepsis	MRCONS (SCCmec non-typable )	CIP,G,CX,VA,LZ
Blood	None	Sepsis, CKD, VAP, myocardial infraction	MRCONS (SCCmec non-typable)	CIP,G,VA,LZ
Blood	aap	Sepsis, urosepsis, DM	MRCONS (SCCmec type III)	G,VA,LZ
Staphylococcus saprophyticus				
Blood	None	CAD, type 2 respiratory failure, bronchiectasis, sepsis	MRCONS (SCCmec type I)	G,CIP,VA,LZ
Staphylococcus arlattae				
Blood	None	Sepsis, CKD, DM, HTN, CAD	MSCONS	CIP, G, CTX, VA, LZ
Blood	None	DCLD, HTN, sepsis	MRCONS (SCCmec type I)	E, CD, CIP, G, VA, LZ
Staphylococcus capitis				
Blood	None	HTN, CKD, post renal transplant graft rejection, sepsis	MSCONS	A,E,CD,CIP,G,CX,VA,LZ
Staphylococcus sciuri				
Blood	None	Sepsis, CKD, CAD, DM, HTN	MSCONS	E, CD, CIP, CXM, G, VA, LZ, TEI
